# Time and Media‐Dependent Modulation of Sperm Capacitation in Red‐Rumped Agouti (*Dasyprocta leporina* Linnaeus, 1758)

**DOI:** 10.1002/mrd.70056

**Published:** 2025-09-02

**Authors:** Lhara Ricarliany Medeiros de Oliveira, Leonardo Vitorino Costa de Aquino, Antonia Beatriz Mendonça Pereira, Ana Lívia Rocha Rodrigues, Luanna Lorenna Vieira Rodrigues, Luana Grasiele Pereira Bezerra, Romário Parente dos Santos, Moacir Franco de Oliveira, Alexandre Rodrigues Silva, Alexsandra Fernandes Pereira

**Affiliations:** ^1^ Laboratory of Animal Biotechnology Federal Rural University of Semi‐Arid Mossoró Brazil; ^2^ Laboratory of Animal Germplasm Conservation Federal Rural University of Semi‐Arid Mossoró Brazil; ^3^ Laboratory of Applied Animal Morphophysiology Federal Rural University of Semi‐Arid Mossoró Brazil

**Keywords:** BSA supplementation, calcium chloride, conservation biology, epididymal sperm, rodent reproduction

## Abstract

The increasing focus on understanding spermatozoa mechanisms in rodents aims to enhance reproductive stability and support conservation efforts, particularly for ecologically significant and declining species like the red‐rumped agouti. We aimed to evaluate the interaction between capacitation media and time, testing BSA concentrations—low (4 mg/mL) and high (15 mg/mL)—with or without 2 mM calcium chloride (CaCl_2_) across three time points. Epididymal sperm were distributed into five groups: without capacitation agent (WCA), high BSA (HBSA), high BSA with CaCl_2_ (HBCa), low BSA (LBSA), and low BSA with CaCl_2_ (LBCa), each incubated for 1, 3, or 6 h. Total sperm motility was significantly higher only in media with CaCl_2_. However, only LBCa maintained high progressive motility. LBCa and HBCa maintained membrane integrity, mitochondrial functionality, and reduced reactive oxygen species levels, but only LBCa increased glutathione levels. HBSA, HBCa, LBSA, and LBCa improved the sperm capacitation rate, but LBCa yielded the highest proportion of capacitated sperm and acrosome‐reacted cells after 6 h. Hyperactivation rates were higher in both LBCa and HBCa after 6 h. Therefore, the optimal condition for red‐rumped agouti sperm capacitation is a low BSA concentration (4 mg/mL) supplemented with 2 mM calcium chloride following a 6 h incubation.

## 1 Introduction

Efforts have been made to elucidate the complexities of spermatozoa in rodents, focusing on optimizing collection methods (Castelo et al. 2023), selecting viable cells (Omid Banafshi et al. 2021), and enhancing their use in assisted reproductive techniques, such as in vitro fertilization (IVF) (Horie et al. 2015). A critical step in this process is ensuring that the male gametes are capacitated and capable of fertilizing the oocyte. Sperm capacitation involves intricate modifications, such as influx of bicarbonate (HCO_3_^−^) and calcium (Ca^2+^) ions into the cell, efflux of cholesterol from the plasma membrane and kinase activation (Nicolli and Cesari 2023). These molecular cascades trigger changes in mitochondrial activity, necessary for altering motility patterns (flagellum hyperactivation), and prepare the sperm for the acrosome reaction (Balestrini et al. 2024).

Data on the specific requirements for sperm capacitation for wild rodents like the red‐rumped agouti (*Dasyprocta leporina*) remain scarce. Optimizing this reproductive phase in these animals substantially enhances their reproductive efficiency by improving the quality of their biological material for biotechnological applications. Such advancements directly support conservation efforts, especially considering the species' ecological significance as a keystone specie and primary seed disperser (Mittelman et al. 2021) alongside the concerning decline of their populations throughout the Americas, with some regions even reporting local extinction in the wild (Zucaratto et al. 2015). To overcome this challenge, phylogenetically proximate species to the red‐rumped agouti, such as the guinea pig (*Cavia porcellus*), can serve as viable models due to similarities in sperm morphophysiology (Mollineau et al. 2008; Gallardo et al. 2002).

Initial research in guinea pigs demonstrated that outcomes similar to those observed in the female reproductive tract can be achieved using minimal capacitating medium (MCM) supplemented with specific capacitation agents, primarily calcium chloride (CaCl_2_) (Ramírez‐Ramírez et al. 2020). Chen et al. (2009) and Ni et al. (2009) underscored the pivotal role of these ions (Ca^2+^ and Cl^−^) in guinea pig epididymal sperm. In their initial experiment, they maintained a consistent concentration of bicarbonate ions (HCO_3_^−^) while excluding chloride ions (Cl^−^), obtaining a notable 30% decrease in capacitation rates compared to the control (with Cl^−^). In their subsequent trial, the omission of calcium ions (Ca^2+^) from the medium led to a 15% decline in the rate of acrosomal reactions and hyperactivated motility.

Bovine serum albumin (BSA) is another crucial modulatory agent for achieving optimal results in sperm capacitation in cavid rodents, inducing phospholipid rearrangement and preparing spermatozoa to penetrate oocyte before fusion (Mohanty et al. 2024). Despite its critical function, the optimal concentration of this compound remains variable across different rodents. In guinea pig, a low concentration of 4 mg/mL BSA was used in association with 2 mM Ca^2+^, achieving a satisfactory capacitation rate of approximately 60% (Ni et al. 2009). In contrast, Hirose et al. (2020) used a higher profile of BSA (15 mg/mL) in association with 3.4 mM Ca^2+^, obtaining excellent results in Syrian hamster (*Mesocricetus auratus*), reaching rates above 77%. Therefore, the requirement for this protein appears to be intrinsically linked to a species‐specific determinant, requiring an evaluation to ascertain its efficacy and optimal activity in red‐rumped agoutis.

The duration of exposure within the capacitating environment directly impacts the efficacy of this process in addition to the specific protein and ionic requirements for sperm capacitation. In *Cavia porcellus*, when using 1.71 mM CaCl_2_, different authors achieved rates from 34.2% (Delgado‐Buenrostro et al. 2016) to 80% (Cordero‐Martínez et al. 2018), increasing only the sperm exposure periods in the capacitation medium from 1 to 2 h, respectively. Some authors even propose an extended capacitation period in conjunction with IVF to maximize the potential of these spermatozoa, recommending durations of up to 6 h in guinea pigs (Chen et al. 2009; Ni et al. 2009).

Therefore, this study was designed to investigate the interaction between the capacitation medium and incubation time on the viability, functionality, and capacitation rate of red‐rumped agouti epididymal sperm. This was achieved by comparing the MCM supplemented with differing concentrations of BSA—a higher (15 mg/mL) or lower (4 mg/mL) profile—with or without the addition of 2 mM CaCl_2_ across varying exposure times (1, 3, or 6 h).

## 2 Materials and Methods

All experiments were approved by the Animal Ethics Committee of the Federal Rural University of Semi‐Arid (no. 20/2021) and Chico Mendes Biodiversity Conservation (ICMBio, no. 76655‐1). The chemicals used in this study were purchased from Sigma‐Aldrich Chemical Co. (St. Louis, MO, USA) except where mentioned otherwise.

### 2.1 Animals and Epididymal Sperm Recovery

The males used in the study were kept at the Center of Multiplication of Wild Animals (CEMAS/UFERSA). They were fed commercial rabbit food and supplied with drinking water ad libitum, using a 12‐h natural photo period. Sexually mature red‐rumped agouti were used for spermatozoa collection (six replicates total). A within‐subjects experimental design was employed, in which spermatozoa collected from each individual (*n* = 6) were equally distributed among the experimental groups per replicate. This design minimized interindividual variability and optimized the use of a limited number of wild animals, while maintaining statistical robustness, as each subject served as its own control.

The agoutis were pre‐medicated with ketamine (15 mg/kg, Ketalar, Pfizer, SP, Brazil) and xylazine (1 mg/kg; Rompun, Bayer, SP, Brazil). Following a 15‐min interval, anesthesia was induced via intramuscular administration of sodium thiopental at 50 mg/kg (Thiopentax; Cristalia, Sao Paulo, SP, Brazil). Subsequently, the animals were euthanized via intravenous administration of potassium chloride at 1 mL/kg (Equiplex, Goiania, GO, Brazil) (Castelo et al. 2015).

The testes‐epididymis was recovered and transported to the laboratory in heated saline solution (37°C, NaCl 0.15 M). The cauda region was excised and the epididymal spermatozoa were obtained by retrograde flushing with 1.0 mL of the heated saline solution (Castelo et al. 2015). The recovered samples were maintained in a water bath at 37°C during initial evaluations. Macroscopic characteristics such as appearance and color were assessed visually. The pH was measured using appropriate pH indicator strips. Sperm vigor was evaluated under a light microscope on a glass slide and scored on a scale from 0 to 5. Sperm concentration was determined using a Neubauer chamber under conventional light microscopy (Castelo et al. 2015).

### 2.2 Sperm Processing and Capacitation Protocols

Spermatozoa were diluted to a concentration of 100 × 10^6^ sperm/mL in minimum capacitation medium (MCM: 105.8 mM NaCl, 25 mM NaHCO_3_, 5.56 mM glucose, 21.6 mM Na lactate, 25 mM HEPES, 0.25 mM sodium pyruvate, 10 µg/mL phenol red, and 1% antibiotic antimycotic solution) (Cañón‐Beltrán et al. 2023). The most viable gametes were selected using a simple centrifugation protocol, where a 1:1 solution with spermatozoa and MCM was placed in a 15 mL plastic tube and washed twice (300 × *g* for 3 min, room temperature). Finally, the supernatant was discarded, and the cells were suspended in 200 µL MCM for analysis (control group) or capacitation (de Oliveira et al. 2025).

**Figure 1:**
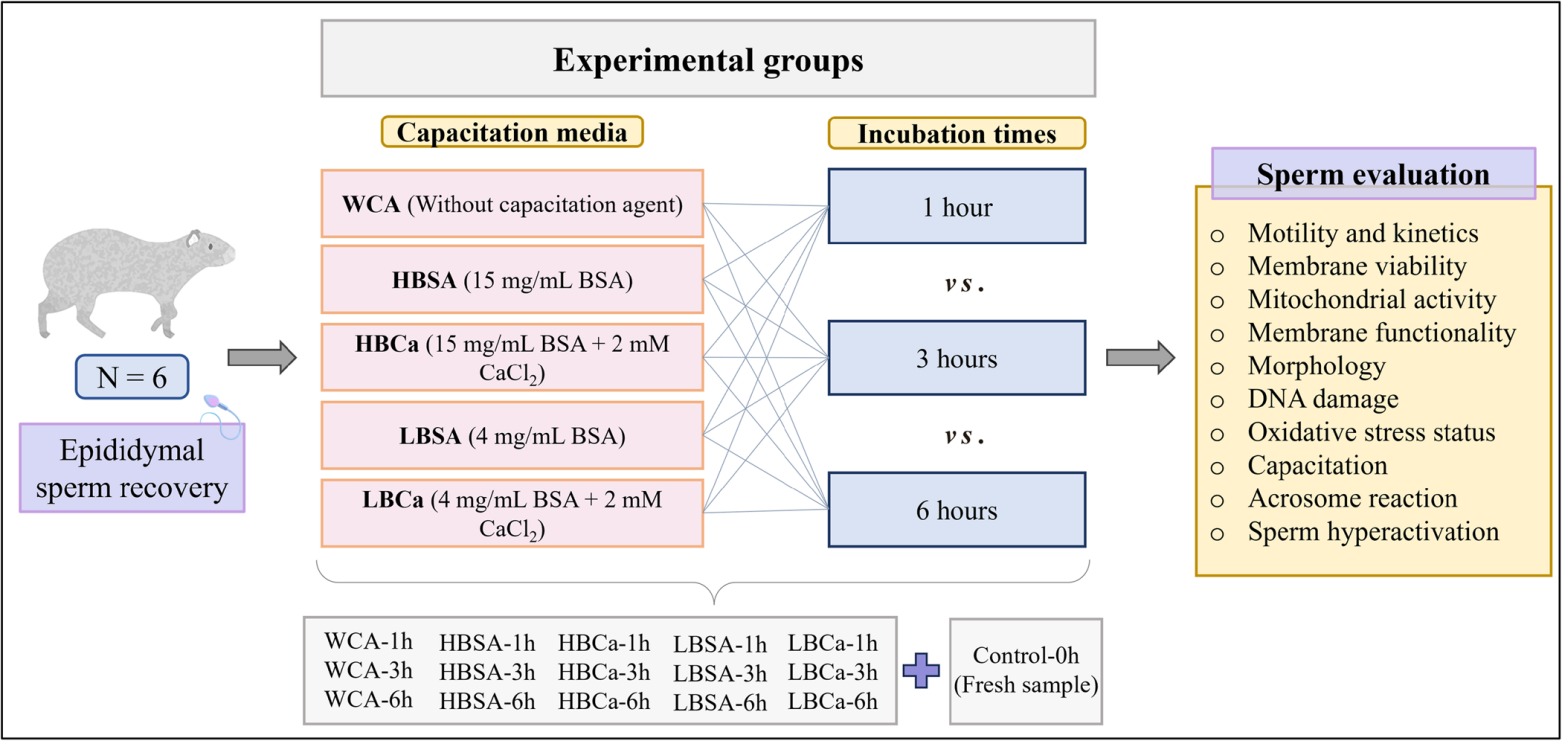
Experimental design for analysis of the interaction between different medium supplementation and incubation times on the viability rate, functionality, and capacitation of epididymal spermatozoa of red‐rumped agouti.

The samples were destined for capacitation at a concentration of 20 × 10^6^ spermatozoa/mL in MCM medium added with capacitation agents. The process was conducted in a controlled atmosphere at 38.5°C with 6.5% CO_2_ (de Oliveira et al. 2025). The spermatozoa were incubated in five different microtubes with capacitation media (Figure 1): without capacitation agents (WCA), 15 mg/mL BSA (HBSA), 15 mg/mL BSA with 2 mM CaCl_2_ (HBCa), 4 mg/mL BSA (LBSA), and 4 mg/mL BSA with 2 mM CaCl_2_ (LBCa). The influence of incubation time was evaluated in a controlled atmosphere for 1, 3, and 6 h for all treatments.

A nonselected‐or‐capacited group with samples immediately after collection in saline solution (NaCl 0.15 M) was analyzed to measure sperm health after epididymal recovery. Moreover, all groups were evaluated by a range of analyses, encompassing sperm kinetics, membrane integrity, normal mitochondrial activity, membrane functionality, morphology, DNA integrity, oxidative stress status, capacitation, acrosome reaction, and flagellar hyperactivation.

### 2.3 Sperm Kinetic Parameters

The samples were analyzed using computer‐assisted sperm analysis (CASA, IVOS 7.4 G; Hamilton‐Thorne Research, MA, USA) with settings previously described for red‐rumped agouti: temperature, 37°C; straightness threshold, 30%; minimum contrast, 45; low‐velocity average pathway (VAP) cutoff of 10 m/s; and medium VAP cutoff of 30 m/s (Castelo et al. 2015). Five independent and nonconsecutive microscopic fields were selected for scanning. The parameters analyzed were total motility (%), progressive motility (%), average pathway velocity (VAP, μm/s), straight‐line velocity (VSL, μm/s), curvilinear velocity (VCL, μm/s), amplitude of lateral head displacement (ALH, μm), beat/cross‐frequency (BCF, Hz), straightness (STR, %), and linearity (LIN, %). The sperm population was subdivided into rapid, medium, and static (%).

The percentage of spermatozoa showing signs of motility hyperactivation (HYP) was determined based on the methodology of Cañón‐Beltrán et al. (2023) with minor alterations. The data obtained from the CASA analysis was restructured to identify the mean of the top 10% of spermatozoa exhibiting the highest VCL and ALH across all groups. For this experiment with red‐rumped agouti sperm the values were: VCL = 65 µm/s and ALH = 5.0 µm/s. Thus, we defined spermatozoon with VCL > 65 µm/s and ALH > 5 µm/s as showing hyperactive‐like motility.

### 2.4 Spermatozoa Membrane Integrity and Mitochondrial Activity

An aliquot of spermatozoa was incubated at 37°C with three fluorescent probes: 40 µg/mL Hoechst 33342 (Molecular Probes, Eugene, OR, USA) and 0.5 mg/mL propidium iodide (Thermo Fisher Scientific, Whaltam, MA, USA) for 5 min, followed by 500 nM CMXRos (Mito Tracker Red, F‐7512, Molecular Probes, Eugene, OR, US) for 8 min. For each experimental group per replica, 100 cells were analyzed using fluorescence microscopy (400×; Olympus BX51TF, Tokyo, Japan) (Celeghini et al. 2007). Spermatozoa displaying a blue‐stained head (Hoechst 33342), no red nuclear staining (PI‐negative), and a red fluorescence in the midpiece (CMXRos‐positive), were classified as viable, with intact plasma membranes and active mitochondria (Santos et al. 2023).

### 2.5 Sperm Membrane Functionality

A hypoosmotic swelling test (HOST) was performed using a solution composed of distilled water (0 mOsm/L) and a sodium citrate and fructose solution (50 mOsm/L). Aliquots of 5 μL containing epididymal spermatozoa were mixed with 45 μL of the hypoosmotic solution and incubated in a dry bath at 37°C for 40 min. Subsequently, the samples were evaluated using a phase‐contrast light microscope (400× magnification, 100 cells per group/per replica). Spermatozoa exhibiting a swollen coiled tail were classified as possessing functional membranes (Dantas et al. 2022).

### 2.6 Sperm Morphology Evaluation

For morphology parameters, a 10 µL aliquot of the sperm samples was fixed and stained with a formaldehyde‐Bengal rose solution (Cromato) and viewed under a light microscope (1000×; 100 cells per group/per replica). They were classified into two categories: normal or abnormal morphology. The abnormalities were also classified as presenting head, middle piece, or tail defects (Silva et al. 2011).

### 2.7 Spermatozoa DNA Damage

Spermatozoa samples were smeared onto glass slides and air‐dried for approximately 2 min. The slides were then fixed in Carnoy's solution for 3 h, rinsed, and air‐dried at room temperature. Subsequently, the slides were incubated for 25 min in a buffer solution comprising 15 mM Na_2_HPO_4_ and 80 mM citric acid (pH 2.5) at 75°C. The smears were stained with acridine orange (0.2 mg/mL) for 10 s, washed with distilled water, and covered with a coverslip while still wet. A total of 100 cells were evaluated per group/per replica using fluorescence microscopy (400× magnification; Olympus BX51TF, Tokyo, Japan). Sperm heads with intact (double‐stranded) DNA exhibited green fluorescence. In contrast, those with denatured or single‐stranded DNA displayed yellow, orange, or red fluorescence, indicative of progressively higher levels of damage (Tomov et al. 2020).

### 2.8 Sperm Capacitation and Acrosome Reaction (AR) Analysis

Sperm capacitation was evaluated using chlortetracycline (CTC) staining, according to the method described by Cordero‐Martinez et al. (2018), with minor modifications. Initially, 40 μL of the sperm suspension was diluted (1:1) with 40 μL of CTC staining solution and incubated for 10 min. The CTC solution comprised of 750 mM CTC in 130.0 mM NaCl, 5 mM cysteine, and 20 mM Tris–HCl (pH 7.8). The samples were washed in MCM (100 × g for 3 min), and slides were mounted with the sperm suspension and observed with fluorescence microscopy (1000×). The sperm were characterized (100 cells per group/per replicate) by the fluorescence pattern described. The F pattern (non‐capacitated) displayed a pale fluorescence in the head, B pattern (capacitated) showed a strong fluorescence in the apical acrosome region, and for AR pattern (acrosome reacted), sperm exhibited no acrosome and showed intense fluorescence on the equatorial segment (Cordero‐Martinez et al. 2018).

### 2.9 Sperm Oxidative Stress Evaluation

The levels of reactive oxygen species (ROS) and intracellular glutathione (GSH) were quantified using 10 µM 2’,7’‐dichlorodihydrofluorescein diacetate (H_2_DCFDA; Invitrogen, Carlsbad, CA, USA) and 10 µM 7‐amino‐4‐chloromethylcoumarin (CellTracker Blue; Invitrogen, Carlsbad, CA, USA), respectively. Spermatozoa were incubated in the dark for 30 min at 37°C in a dry bath. Subsequently, the samples were washed twice (500 × *g*, 5 min), resuspended in phosphate‐buffered saline (PBS), mounted in glass slides, and analyzed using fluorescence microscopy (400 × magnification). Fluorescence intensity were quantified with ImageJ software (National Institutes of Health, Bethesda, Maryland, USA) to determine ROS and GSH levels. The Nonselected‐or‐capacited group served as a calibrator. The measured values of each treatment were normalized to the calibrator mean to generate relative expression levels (arbitrary fluorescence units, AFU) (Santos et al. 2023). Following the collection of AFU data, the ROS/GSH ratio was calculated to ascertain the oxidative status of the samples.

### 2.10 Statistical Analysis

The results were expressed as mean ± standard error (one male/one replicate). Normality of residuals was assessed using the Shapiro‐Wilk test, and variance homogeneity was evaluated via the Bartlett test. Percentile data were subjected to angular transformation [ARC‐Sine (√x/100)] to approximate a normal distribution and ensure stability. Due to repeated measures over time, the analyzed parameters (sperm kinetics and motility, membrane integrity, mitochondrial activity, membrane functionality, morphology, DNA integrity, oxidative stress status, capacitation, acrosome reaction, and flagellar hyperactivation) were examined using a mixed linear model (mixed effect analysis procedure). This model included the main effects, different BSA profiles with or without CaCl_2_ (HBSA vs. HBCa vs. LBSA vs. LBCa), the incubation interval (1 h vs. 3 h vs. 6 h), and their interactions. The Tukey test was employed for mean comparison when significant effects were detected in the variance analysis (*F*‐test). All statistical analyses were performed using GraphPad Prism version 8 for Windows (GraphPad Software Inc. San Diego, CA, USA), with significance set at *p* < 0.05.

## 3 Results

All epididymal flushed samples exhibited a whitish color, with an average pH of 7.0 and a vigor score of 4.0 on a 0–5 scale. The mean sperm concentration was 252.3 × 10^6^ spermatozoa/mL, with a range from 179 to 290 × 10^6^ spermatozoa/mL.

The statistic test for mixed effects showed a significant result of the main effects and the interaction (treatment × time) on the following sperm parameters: kinetics (BCF, STR, and LIN), normal morphology and tail defects, capacitation rate, acrosome reaction, ROS and GSH levels, ROS/GSH proportion, and hyperactivation analysis. Furthermore, the other variables exhibited no interaction effects, with observed differences attributable solely to the treatment conditions or the incubation time.

### 3.1 Red‐Rumped Agouti Epididymal Spermatozoa Characteristics

The results for the red‐rumped agouti Nonselected‐or‐capacited samples immediately after epidydimal recovery are represented in Table 1 and Table 2. Sperm kinetic analysis (Table 1) demonstrated a total motility of 91.8% and a progressive motility of 43.3%, reflecting the overall good quality of the samples. Additional data from the remaining analyses are summarized in Table 2. The mitochondrial activity and plasma membrane integrity data revealed that 22.1% ± 13.1% of spermatozoa exhibited an intact membrane and normal mitochondrial activity, while 43.8% ± 13.7% showed no mitochondrial activity. Additionally, 3.0% ± 1.5% had compromised membrane integrity, and 31.0% ± 13.3% were completely damaged. In summary, 66.0% ± 14.8% of the sperm cells showed mitochondrial activity loss, and 25.1% ± 12.5% exhibited plasma membrane damage. Furthermore, the membrane functionality rate of these samples was 48.6% ± 10.4%.

**Table 1:** Computer‐aided sperm analysis of fresh (0 h) noncapacitated samples of red‐rumped agouti epididymal sperm.

CASA parameters	(Mean ± SE)
Total motility (%)	91.8 ± 3.3
Progressive motility (%)	43.3 ± 12.6
VAP (µm/s)	80.8 ± 19.1
VSL (µm/s)	68.9 ± 18.0
VCL (µm/s)	111.6 ± 20.3
ALH (µm/s)	4.9 ± 0.1
BCF (Hz)	37.0 ± 1.1
STR (%)	76.1 ± 3.7
LIN (%)	51.8 ± 6.1
Rapid (%)	51.3 ± 14.8
Medium (%)	38.0 ± 11.3
Static (%)	10.6 ± 3.7

Abbreviations: ALH, amplitude of lateral head; BCF, beat cross frequency; LIN, linearity; SE, standard error; Sperm population, rapid, medium or static; STR, straightness; VAP, velocity average pathway; VCL, curvilinear velocity; VSL, velocity straight line.

**Table 2:** Epidydimal sperm parameters of red‐rumped agouti.

Nonselected‐or‐capacited samples data							
**Mitochondrial activity and plasma membrane integrity (%)**							
*IPM (+)* *MF (+)*	*IPM (+)* *MF (‐)*		*IPM (−)* *MF (+)*	*IPM (−)* *MF (−)*	*IPM/TOTAL*		*MF/TOTAL*
22.1 ± 13.1	43.8 ± 13.7		3.0 ± 1.5	31.0 ± 13.3	66.0 ± 14.8		25.1 ± 12.5
**Host (%)**							
*Membrane function (+)*				*Membrane function (−)*			
48.6 ± 10.4				51.4 ± 10.4			
**Sperm morphology (%)**							
Normal				*Abnormal*			
89.8 ± 1.6				10.2 ± 1.6			
**Sperm morphological defects (%)**							
*Head*			*Middle piece*		*Tail*		
3.3 ± 6.0			1.8 ± 0.8		5.0 ± 1.2		
**Oxidative stress (AFU)**							
*ROS levels*			*GSH levels*		*ROS/GSH ratio*		
1.00 ± 0.2			1.00 ± 0.2		1.00 ± 0.2		
**Sperm capacitation (%)**							
*Capacited sperm*			*Acrosome reaction*		*Hyperactivation*		
20.2 ± 2.4			16.3 ± 1.9		33.0 ± 0.2		
**DNA damage (%)**							
*Intact DNA*		*Low damage*		*Medium damage*		*High damage*	
90.0 ± 2.6		2.8 ± 1.0		4.3 ± 1.5		2.8 ± 1.1	

*Note:* Mean ± standard error.

Abbreviations: −, absence; +, presence; AFU, arbitrary fluorescence units; HOST, hypoosmotic test; IPM, intact plasma membrane; MF, mitochondrial function.

Regarding spermatozoa morphology, 89.8% ± 1.6% presented normal characteristics, and the abnormal sperm presented defects in the head (3.3% ± 6.0%), middle piece (1.8% ± 0.8%), and tail (5.0% ± 1.2%). The DNA damage rate results for the non‐capacitated sperm: intact DNA (90.0% ± 2.6%), low damage (2.8% ± 1.0%), medium damage (4.3% ± 1.5%), and high damage (2.8% ± 1.1%). Additionally, the oxidative stress parameters were as follows: ROS levels (1.00 AFU ± 0.2), GSH levels (1.00 AFU ± 0.2) and the ROS/GSH ratio was 1.00 AFU ± 0.2.

Finally, for the capacitation analysis, only 20.2% ± 2.4% sperm were spontaneously capacitated after recovery, and 16.3% ± 1.9% had a spontaneous acrosome reaction. The percentage of fresh spermatozoa with hyperactive flagella was 33.0% ± 0.2%.

### 3.2 Effects of Media × Incubation Time on Sperm Morphology and Functionality

Sperm kinetics (Figure 2) showed differences in all parameters (*p* < 0.05), except ALH (*p* > 0.05). The total motility was higher in media with CaCl_2_ regardless of time and BSA profile (*p* < 0.05), ranging from 81.3% ± 4.1% (1 h) to 50.0% ± 10.6% (6 h) for the HBCa and 86.5% ± 4.4% (1 h) to 74.8% ± 5.7% (6 h) for LBCa (Figure 2A). The progressive motility results were similar, with better results only in CaCl_2_ groups (*p* < 0.05); however, only LBCa maintained the progression high for 1 h (49.8% ± 8.0%), 3 h (41.8% ± 6.7%), and 6 h (43.5% ± 4.2%) (Figure 2B, *p* < 0.05). For VAP, VSL, and VCL (Figure 2C–E), all treatments presented similar values at 1 h (*p* > 0.05), but HBCa and LBCa maintained the average velocity also at 3 h and 6 h (*p* < 0.05). BCF, STR, and LIN (Figure 2G–I) remained stable in all groups during the 6 h incubation (*p* > 0.05), except for HBSA, which showed low values after 1 h (*p* < 0.05). Finally, the rapid sperm population (Figure 2J) was higher in media with CaCl_2_ regardless of incubation time (*p* < 0.05). Still, the static population (Figure 2L) was higher in HBCa compared to LBCa after 6 h (*p* < 0.05).

Figure 2Efficiency of different capacitation protocols of epididymal sperm kinetic parameters of red‐rumped agouti. (A) Total motility rate. (B) Progressive motility rate. (C) Average pathway velocity. (D) Straight‐line velocity. (E) Curvilinear velocity. (F) Amplitude of lateral head displacement. (G) Beat/cross‐frequency. (H) Straightness rate. (I) Linearity rate. (J) Rapid sperm population rate. (K) Medium sperm population rate. (L) Static sperm population rate. Letters indicate differences between treatments within the same time. Asterisk indicates differences between times within the same treatment. *p* < 0.05.

**Figure:**
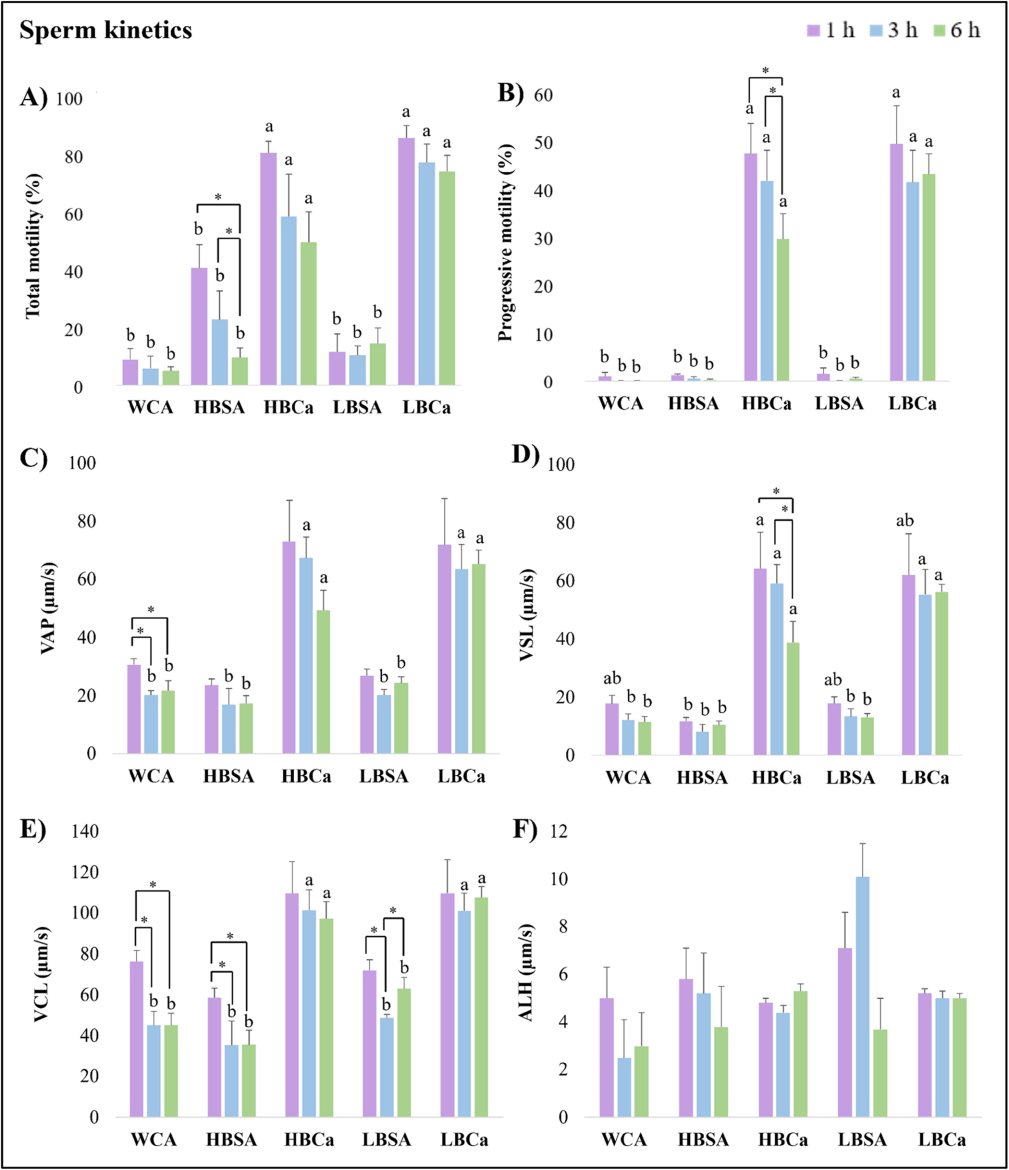

**Figure:**
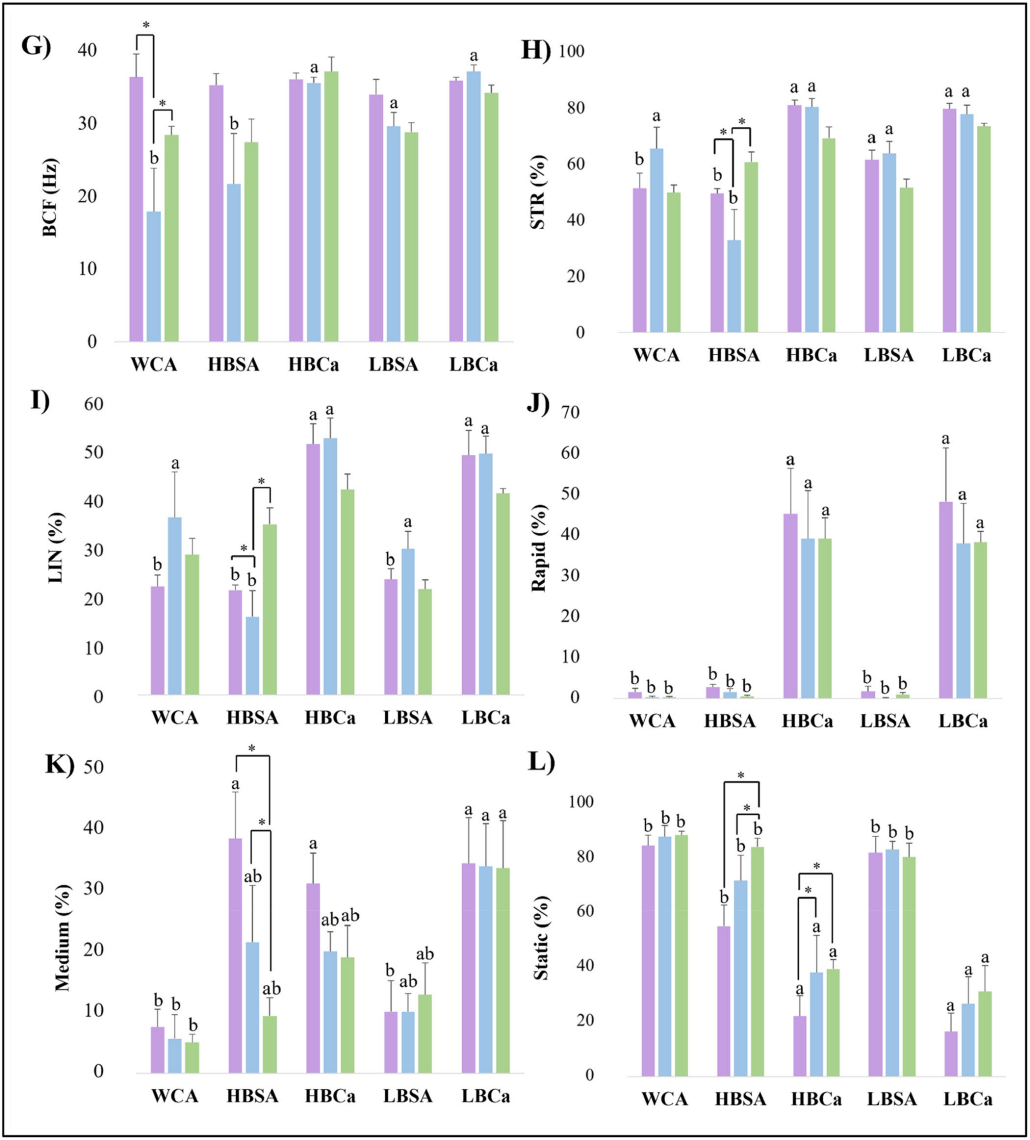

The incubation time did not affect sperm mitochondrial activity and plasma membrane integrity rates (Table 3). However, between treatments, both HBCa and LBCa maintained these parameters (*p* < 0.05). In addition, after 6 h of sperm capacitation, all treatments presented a higher number of sperm cells with absent mitochondrial activity, except HBCa and LBCa (*p* < 0.05). Either time or different capacitation media did not affect membrane functionality (*p* > 0.05) (Table 4).

**Table 3:** Effects of different capacitation conditions on mitochondrial activity and plasma membrane integrity of red‐rumped agouti sperm.

Sperm mitochondrial activity and plasma membrane integrity			
Groups	Capacitation times		
	1 h	3 h	6 h
*Intact membrane + functional mitochondria*			
WCA	20.5 ± 9.4^b^	14.1 ± 10.6^b^	29.5 ± 9.7^a^
HBSA	45.1 ± 7.2^b^	46.5 ± 7.2^ab^	38.3 ± 3.5^a^
HBCa	75.6 ± 4.0^a^	76.8 ± 3.7^a^	70.3 ± 13.3^a^
LBSA	37.3 ± 7.5^b^	33.5 ± 9.5^ab^	37.0 ± 7.1^a^
LBCa	76.8 ± 3.9^a^	69.1 ± 3.7^a^	65.6 ± 12.8^a^
*Intact membrane + nonfunctional mitochondria*			
WCA	44.6 ± 13.2^a^	24.5 ± 10.9^a^	26.0 ± 10.0^b^
HBSA	20.6 ± 11.2^a^	23.3 ± 11.5^a^	31.6 ± 6.5^b^
HBCa	10.1 ± 5.3^a^	7.3 ± 1.5^a^	3.0 ± 1.0^a^
LBSA	40.1 ± 11.0^a^	33.1 ± 11.4^a^	23.0 ± 5.7^b^
LBCa	10.8 ± 4.8^a^	9.8 ± 4.0^a^	9.0 ± 4.4^a^
*Ruptured membrane + functional mitochondria*			
WCA	8.6 ± 5.1^a^	16.0 ± 9.2^a^	0.1 ± 0.1^a^
HBSA	0.5 ± 0.3^a^	1.3 ± 0.6^a^	1.5 ± 0.7^a^
HBCa	0.0 ± 0.0^a^	1.8 ± 1.6^a^	11.1 ± 11.1^a^
LBSA	1.6 ± 0.9^a^	0.6 ± 0.3^a^	2.1 ± 1.1^a^
LBCa	0.3 ± 0.3^a^	0.1 ± 0.1^a^	8.0 ± 8.0^a^
*Ruptured membrane + nonfunctional mitochondria*			
WCA	26.1 ± 7.2^a^	45.3 ± 12.8^a^	44.5 ± 16.4^a^
HBSA	33.6 ± 7.0^a^	28.8 ± 6.5^a^	28.5 ± 6.3^a^
HBCa	14.1 ± 3.7^a^	14.0 ± 2.6^a^	15.5 ± 2.9^a^
LBSA	20.8 ± 8.0^a^	32.6 ± 13.0^a^	37.8 ± 8.7^a^
LBCa	12.0 ± 1.3^a^	20.8 ± 3.7^a^	17.3 ± 6.3^a^
*Intact membrane/total*			
WCA	65.1 ± 11.3^a^	38.6 ± 16.3^a^	55.5 ± 16.6^a^
HBSA	65.8 ± 6.9^a^	69.8 ± 6.8^a^	70.0 ± 7.0^a^
HBCa	85.8 ± 3.7^a^	84.1 ± 3.6^a^	73.3 ± 13.7^a^
LBSA	77.5 ± 8.8^a^	66.6 ± 13.2^a^	60.0 ± 9.2^a^
LBCa	87.6 ± 1.4^a^	79.0 ± 3.8^a^	74.6 ± 14.2^a^
*Functional mitochondria/total*			
WCA	29.1 ± 8.4^b^	30.1 ± 10.9^b^	29.6 ± 9.6^b^
HBSA	45.6 ± 6.9^b^	47.8 ± 7.2^b^	39.8 ± 3.3^b^
HBCa	75.6 ± 4.0^a^	78.6 ± 2.9^a^	81.5 ± 2.6^a^
LBSA	39.0 ± 7.33^b^	34.1 ± 9.3^b^	39.1 ± 7.3^b^
LBCa	77.1 ± 4.0^a^	69.3 ± 3.6^a^	73.6 ± 5.5^a^

*Note:* Mean ± standard error. Superscript letters indicate differences between treatments within the same time point (*p* < 0.05). No differences across time points were detected (*p* > 0.05). WCA: without capacitation agent. HBSA: MCM + 15 mg/mL BSA. HBCa: MCM + 15 mg/mL BSA + 2 mM CaCl_2_. LBSA: MCM + 4 mg/mL BSA. LBCa: MCM + 4 mg/mL BSA + 2 mM CaCl_2_.

**Table 4:** Evaluation of epididymal sperm membrane functionality in red‐rumped agouti after capacitation in different conditions.

HOST (% ± SE)			
Groups	Capacitation times		
	1 h	3 h	6 h
**WCA**	70.8 ± 11.7	79.6 ± 3.7	75.8 ± 1.3
**HBSA**	72.1 ± 6.0	67.0 ± 7.9	72.3 ± 5.0
**HBCa**	88.1 ± 2.4	77.5 ± 5.8	78.5 ± 4.6
**LBSA**	77.6 ± 6.1	69.0 ± 7.0	73.0 ± 7.1
**LBCa**	88.6 ± 1.7	85.3 ± 2.9	84.3 ± 1.5

*Note: p* > 0.05. HBSA: MCM + 15 mg/mL BSA. HBCa: MCM + 15 mg/mL BSA + 2 mM CaCl_2_. LBSA: MCM + 4 mg/mL BSA. LBCa: MCM + 4 mg/mL BSA + 2 mM CaCl_2_.

Abbreviations: HOST, hypoosmotic test; SE, standard error; WCA, without capacitation agent.

**Figure 3:**
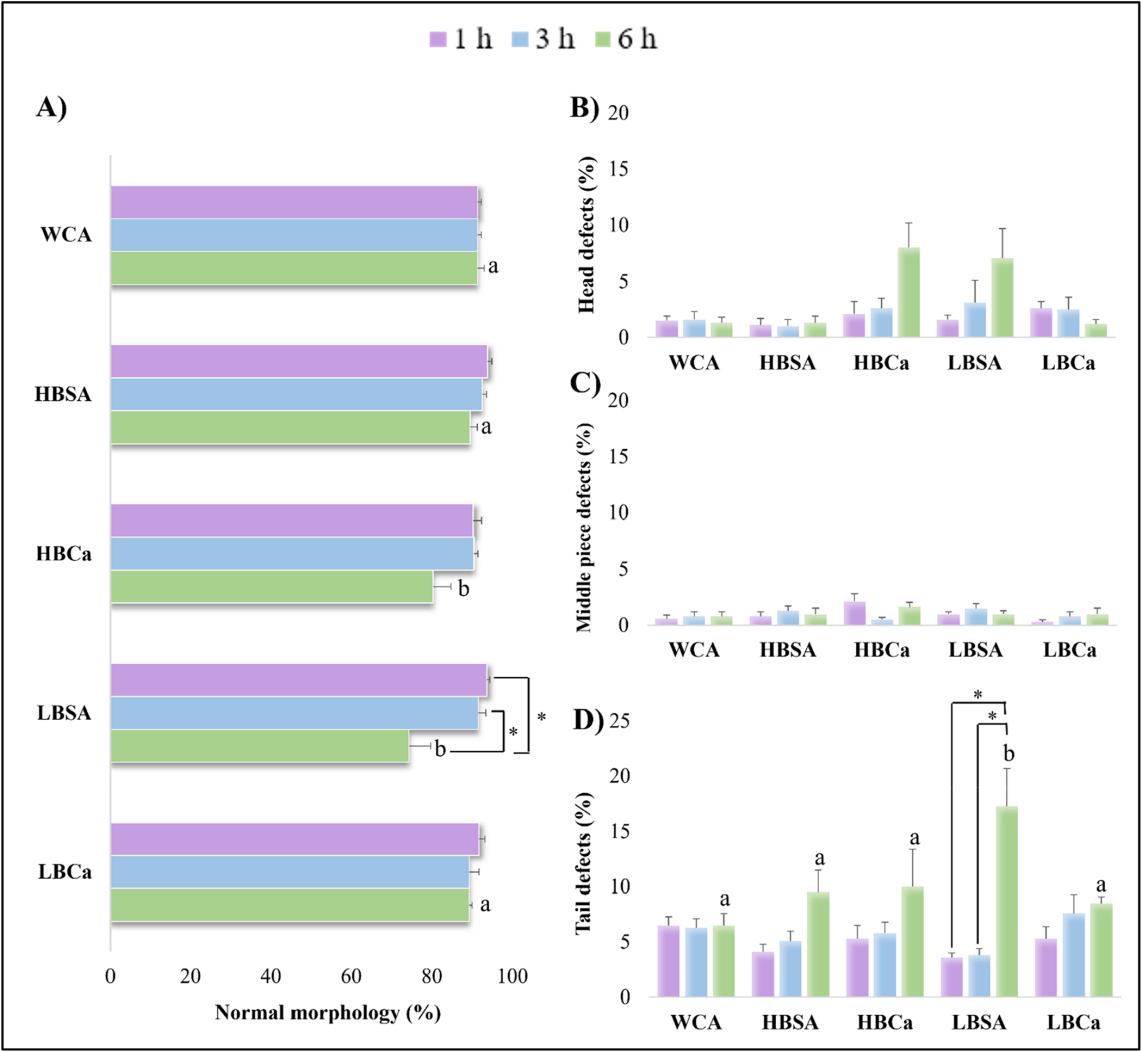
Effects of different capacitation media and incubation times for morphology characteristics of the sperm of red‐rumped agouti. (A) Percentage of the sperm population with normal morphology after capacitation. (B) Rate of head defects in agouti sperm. (C) Middle piece defects rate. (D) Tail abnormalities rate in capacitated sperm. Letters indicate differences between treatments within the same time. Asterisk indicates differences between times within the same treatment. *p* < 0.05.

**Figure 4:**
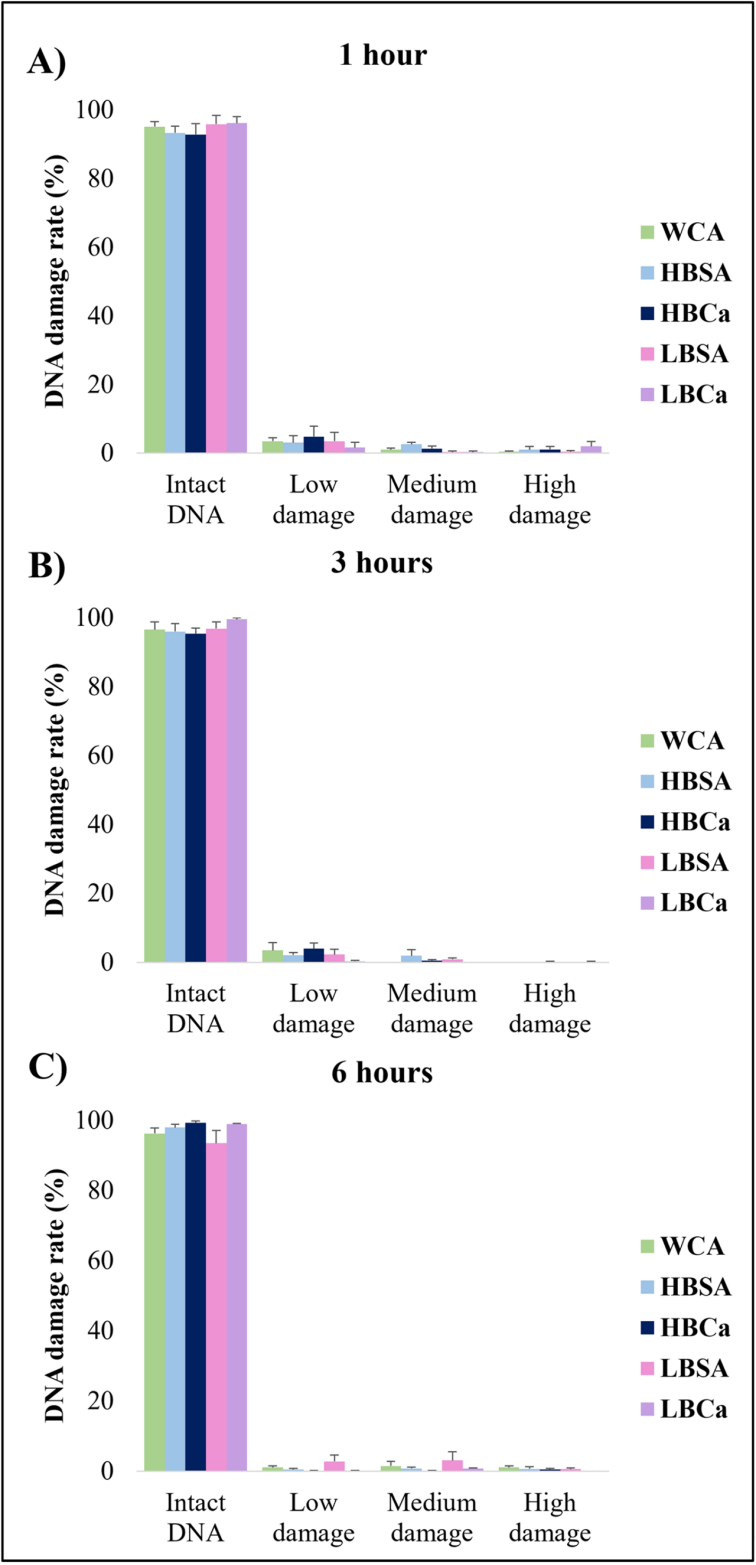
DNA damage index of the sperm of red‐rumped agouti after different capacitation protocols. (A) Percentage of intact, low, medium, and high damage on the DNA strand after 1 h, (B) 3 h, and (C) 6 h of incubation. *p* > 0.05.

Sperm cells presented around 90% normal morphology rates after capacitation (Figure 3) for all treatments for 1 h and 3 h (*p* > 0.05). However, the HBCa and LBSA groups showed a 10%–15% reduction at 6 h (Figure 3A, *p* < 0.05). There were no differences in the head and middle piece (*p* > 0.05) among the morphological abnormalities found, but tail defects (Figure 3D) were more than triple after 6 h in the low BSA without CaCl_2_ (*p* < 0.05). Sperm capacitation conditions in the DNA damage rate affected none of the experimental groups (Figure 4, *p* > 0.05). Even after 6 h of incubation, the rates of intact DNA were above 90% while those of low, medium, and high damage were only 1%–3% of the sperm population (*p* > 0.05).

**Figure 5:**
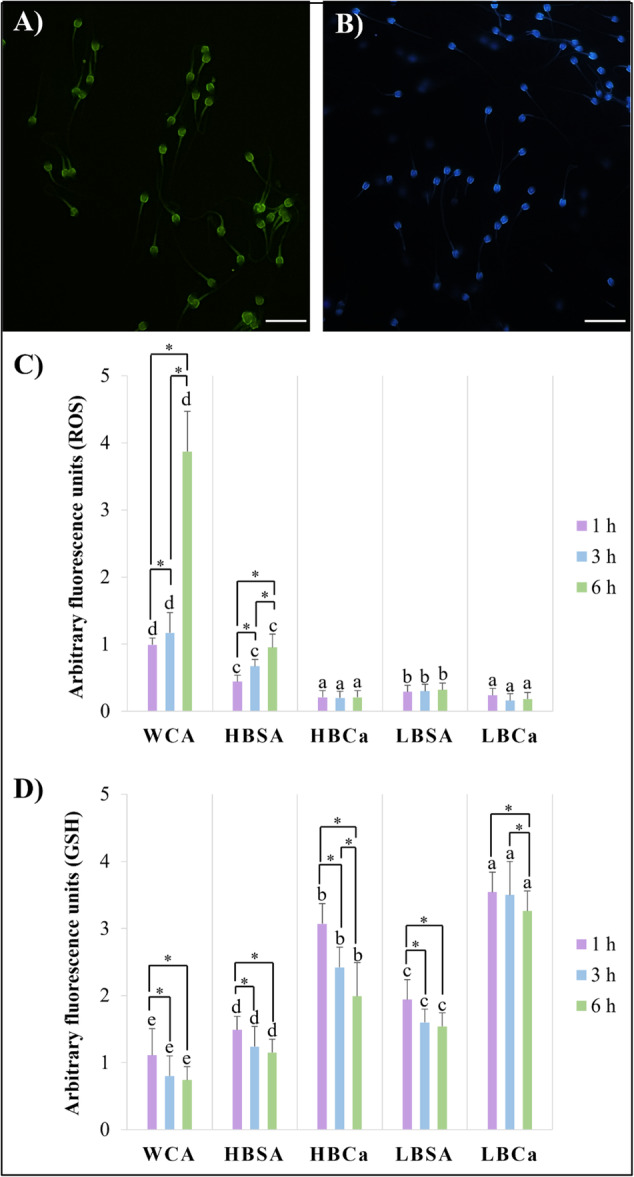
Evaluation of oxidative stress status after different sperm capacitation protocols in red‐rumped agouti. (A) Representative image of spermatozoa stained with H_2_DCFDA for ROS levels evaluation and (B) CellTracker blue for GSH levels evaluation. Scale bar: 20 µM. 40× magnification. (C) ROS levels in capacitated sperm in different media after 1, 3, and 6 h. (D) GSH levels in sperm were capacitated in different medias after 1, 3, and 6 h. Letters indicate differences between treatments within the same time. Asterisk indicates differences between times within the same treatment. *p* < 0.05.

The oxidative stress status after capacitation was determined by the ROS and GSH levels (Figure 5A,B), which showed significant differences between the capacitation media and the incubation times (*p* < 0.05). The sperm cells capacitated with different BSA profiles with CaCl_2_ (HBCa and LBCa) maintained low levels of reactive oxygen species (Figure 5C) during 1 h, 3 h, or 6 h of incubation (*p* < 0.05). They were more efficient than all other groups (*p* < 0.05). WCA and HBSA showed higher ROS levels (*p* < 0.05) that increased significantly over 3 h and 6 h (*p* < 0.05). The LBSA showed higher ROS levels than HBCa and LBCa (*p* < 0.05), but this number did not increase as the hours passed (*p* > 0.05). For the intracellular glutathione (Figure 5D), LBCa presented the highest levels compared to the other treatments (*p* < 0.05), but this value decreased to 6 h (*p* < 0.05). The ROS/GSH proportion (Table 5) showed that LBSA, HBCa, and LBCa presented an almost zero proportion, indicating that low levels of ROS were always linked to high levels of GSH, while the WCA and HBSA showed significantly higher numbers for this proportion (*p* < 0.05). In addition, the WCA and HBSA proportion increased over time (*p* < 0.05), while the others managed to maintain their numbers even after 6 h of capacitation (*p* > 0.05).

**Table 5:** ROS/GSH proportion of red‐rumped agouti spermatozoa cells after different capacitation treatments and incubation times.

ROS/GSH proportion (mean ± SE)			
Groups	Capacitation times		
	1 h	3 h	6 h
**WCA**	0.93 ± 0.1^cA^	1.50 ± 0.2^cB^	5.41 ± 0.6^cC^
**HBSA**	0.30 ± 0.1^bA^	0.57 ± 0.1^bA^	0.85 ± 0.1^bB^
**HBCa**	0.07 ± 0.1^aA^	0.09 ± 0.1^aA^	0.11 ± 0.1^aA^
**LBSA**	0.15 ± 0.1^aA^	0.18 ± 0.1^aA^	0.21 ± 0.1^aA^
**LBCa**	0.07 ± 0.1^aA^	0.05 ± 0.1^aA^	0.05 ± 0.1^aA^

*Note:* SE: standard error. *p* < 0.05. Lowercase letters indicate differences between treatments within the same time. Uppercase letters indicate differences between times within the same treatment. WCA: without capacitation agent. HBSA: MCM + 15 mg/mL BSA. HBCa: MCM + 15 mg/mL BSA + 2 mM CaCl_2_. LBSA: MCM + 4 mg/mL BSA. LBCa: MCM + 4 mg/mL BSA + 2 mM CaCl_2_.

**Figure 6:**
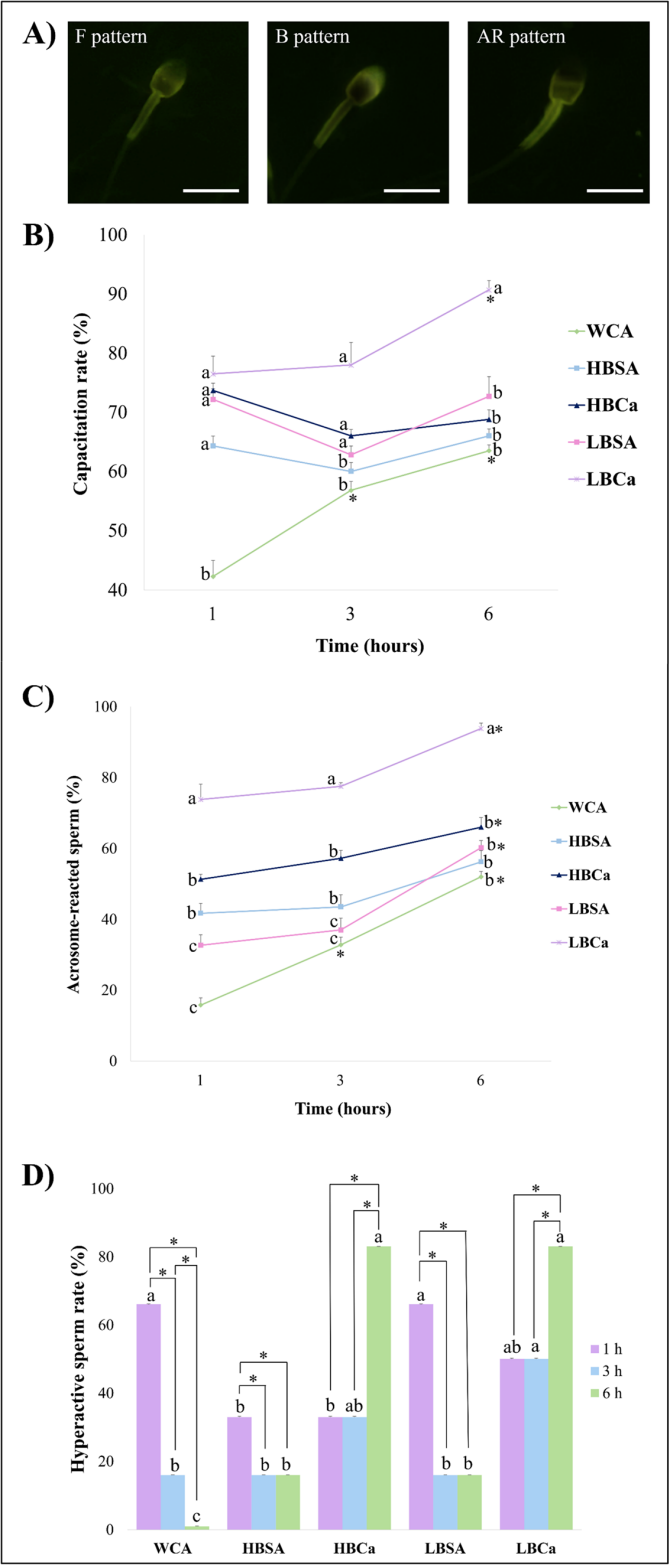
Capacitation, acrosome‐reaction, and hyperactive rates after different treatments for the epididymal sperm of red‐rumped agouti. (A) Representative image of CTC‐stained sperm cells for capacitation and AR evaluation. F pattern (non‐capacitated) displayed a pale fluorescence in the head, B pattern (capacitated) showed a strong fluorescence in the apical acrosome region, and for the AR pattern (acrosome reacted sperm), sperm exhibited no acrosome and showed intense fluorescence on the equatorial segment. Scale bar: 100 µM. 100× magnification. (B) Capacitation rates of agouti sperm after 1, 3, and 6 h in different media. (C) AR rates after different protocols of capacitation in agouti. (D) Hyperactive sperm percentage after treatments and incubation times. Letters indicate differences between treatments within the same time. Asterisk indicates differences between times within the same treatment. *p* < 0.05.

All treatments, besides the WCA, improved sperm capacitation rate (Figure 6A,B) at 1 h (*p* < 0.05). After 3 h, only HBCa, LBSA, and LBCa maintained higher capacitation rates (*p* < 0.05). Finally, after 6 h, the best group for sperm cells was the LBCa, reaching around 90.7% of capacitation (*p* < 0.05). The data for acrosome‐reacted sperm (Figure 6C) showed that the LBCa was superior to all treatments and the peak response occurred after 6 h (*p* < 0.05). In addition, the status of hyperactive sperm (Figure 6D) was higher for both CaCl_2_ groups (HBCa and LBCa) after 6 h (*p* < 0.05), with similar data to both WCA and LBSA at 1 h (*p* > 0.05).

## 4 Discussion

The results of this study showed that a lower BSA concentration (4 mg/mL) associated with 2 mM calcium chloride (LBCa group) during 6 h incubation is the most effective protocol for capacitating epididymal spermatozoa of red‐rumped agouti. This protocol‐maintained sperm progressive motility, membrane viability, morphological integrity, and normal mitochondrial activity while promoting reduced ROS production and elevated GSH levels. Additionally, it yielded a higher proportion of capacitated spermatozoa exhibiting acrosome reaction and hyperactive motility.

The optimal sperm kinetics parameters, including total motility, progressive motility, linearity, and curvilinear velocity, may serve as valuable prognostic indicators of sperm fertilization potential (Vallet‐Buisan et al. 2023). The maintenance of these characteristics in vitro may be directly linked to the environmental conditions in which these cells are found, such as the medium, temperature, and incubation time (Sansegundo et al. 2022). Both groups highlighted in our research (HBCa and LBCa) have two crucial agents for obtaining sperm movement: calcium and BSA.

Calcium (Ca^2+^) is a fundamental messenger that contributes to sperm motility, related to crucial signaling pathways, such as cAMP/protein kinase A and phosphoinositide 3‐kinase signaling (Dcunha et al. 2022). Likewise, Ca^2+^ has already been reported as essential for sperm motility initiation in mammals (Carroll 2001) and is directly linked to the functioning of the CatSper channels, which is most likely involved in the motility process since CatSper mutant mouse sperm cannot penetrate coat‐intact eggs during IVF (Ren et al. 2001). The BSA modulates all these actions since it can induce Ca^2+^ influx in sperm (Xia et al. 2009).

Maintaining membrane integrity was more significant within the HBCa and LBCa groups, regardless of incubation time. This information is relevant because ruptured membranes have previously been associated with DNA fragmentation and chromatin decondensation, indicative of early apoptotic processes (Bloch et al. 2021). Therefore, BSA may have acted protectively, with a rapid membrane binding primarily aiding stabilization through interaction with phospholipids reducing membrane damage (Álvarez‐Rodríguez et al. 2024). Additionally, the calcium has been shown to regulate membrane fluidity and prevent premature destabilization, particularly under capacitating or stress‐inducing conditions (Xue et al. 2025).

In addition, the mitochondrial activity was also normal within the HBCa and LBCa groups. These results corroborate our findings, where the higher capacitation rates also occurred in the HBCa and LBCa groups, corroborating what has already been seen in previous studies that mitochondrial respiration increases during capacitation in mouse sperm (Balbach et al. 2020). However, after 6 h of incubation, all calcium‐free groups significantly reduced mitochondrial activity. This result can be explained by the lack of calcium in the medium since the increase in mitochondrial activity in capacitated sperm parallels the increase in mitochondrial calcium concentration during the capacitation of rodent sperm (Ferreira et al. 2021).

A higher incidence of morphological abnormalities was observed in two specific groups after 6 h of incubation: the group with a high BSA concentration supplemented with calcium (HBCa) and the group with a low BSA concentration without calcium (LBSA). These findings suggest that red‐rumped agouti spermatozoa may be particularly sensitive to both BSA concentration and the duration of exposure. BSA is known to facilitate the removal of cholesterol from the sperm plasma membrane, leading to phospholipid displacement and reorganization, which in turn increases membrane fluidity (Mohanty et al. 2024). While this is a critical step in capacitation, excessive cholesterol efflux—such as that potentially induced by supraphysiological BSA levels in the HBCa group—may destabilize the membrane and compromise sperm structural integrity, especially over prolonged incubation periods.

Additionally, the increased frequency of tail abnormalities in this group may be directly associated with the lower sperm motility parameters observed under the same conditions, as the tail is fundamental for flagellar movement and overall motility (Yogo 2022). This supports the notion that extended exposure to high BSA concentrations, even in the presence of calcium, may lead to detrimental effects on sperm structure and function.

In contrast, the LBSA group, which lacked calcium and was incubated with suboptimal BSA concentration, also showed a high rate of morphological defects. It is plausible that the low BSA concentration was insufficient to adequately trigger capacitation‐related membrane remodeling, and prolonged incubation under these conditions may have resulted in membrane instability or oxidative stress. The absence of calcium may have further exacerbated this vulnerability, given its well‐documented role in maintaining membrane stability and protecting against structural damage (Xue et al. 2025). Notably, these abnormalities were not observed in the LBCa group, suggesting that the presence of calcium may have provided a stabilizing effect, mitigating the adverse outcomes seen in its calcium‐free counterpart.

Several critical biological processes of mammalian spermatozoa are redox regulated, including capacitation, hyperactivation, and acrosomal exocytosis (Bennetts and Aitken 2005). Sperm produce and export ROS to the extracellular environment, most of which are generated by the mitochondria, secondary to the flagellar activity of the cells. The loss of sperm function, that is, the fertilization capacity, could result from the presence of high levels of ROS (Parodi 2021). Only the groups with calcium maintained the lowest ROS levels for 6 h. Since motility generates ROS, it is hypothesized that regulation by calcium reduces excess motility and the general metabolic state of the cells, leading to a reduction of oxidative stress (Parodi 2021). The low ROS production may also be related to higher levels of the cellular antioxidant present, such as intracellular GSH in the LBCa groups, which could have its production facilitated by the protection guaranteed by the adequate concentration of BSA and CaCl_2_ in the medium.

Capacitation is crucial for the sperm to acquire its fertilizing capacity. In guinea pigs, Ca^2+^ Cl^‐^ and BSA are essential to increase capacitation rates, acrosomal reactions, and hyperactivated motility (Chen et al. 2009; Ni et al. 2009). These substances were also essential for red‐rumped agouti in our study. Using a low concentration of 4 mg/mL BSA in association with 2 mM Ca^2+^, we achieved higher capacitation rates of 90.7%, compared with approximately 60% rate in guinea pig with the same media components (Ni et al. 2009).

All treatments except WCA improved capacitation rates after 1 h of incubation. This may be explained by the presence of BSA in the media and the minimal time for this agent to operate, since BSA induces tyrosine phosphorylation by the modulation of protein kinase A (PKA), and the tyrosine phosphorylation is a slow process starting at ~ 45 min of incubation in capacitation media in mouse sperm (Visconti 2009). However, the maximum capacitation rate for red‐rumped agouti was seen after 6 h incubation with the LBCa. These results highlight the species‐specific time necessary for better capacitation. This was previously demonstrated in guinea pig. When using 1.71 mM CaCl_2_, different authors achieved rates from 34.2% (Delgado‐Buenrostro et al. 2016) to 80% (Cordero‐Martínez et al. 2018), increasing the sperm exposure periods in the capacitation medium of choice from 1 to 2 h, respectively.

Another significant result was the maximum acrosome‐reacted sperm rate after 6 h in LBCa. This phenomenon is necessary to penetrate the zona pellucida so the sperm can fuse with the oocyte. The AR is activated by calcium ions, as demonstrated in mice, the AR and the ability to achieve fertilization do not occur in Ca^2+^ free capacitation media (Navarrete et al. 2015). In addition, a higher rate of AR before fertilization is desired in rodents, as seen by Jin et al. (2011) in mouse when investigating the effect of AR on IVF outcomes, finding that the spermatozoa that began the AR before reaching the zona penetrates the zona and fused with the oocyte's plasma membrane. Also, the most fertile sperm cells underwent an AR before reaching the zona pellucida of *cumulus*‐enclosed oocytes.

Finally, the higher hyperactivation motility rates in the LBCa and HBCa groups could be related to the calcium in the media. Since an increase in the intracellular calcium level (higher than 200 nM) is needed for the spermatozoa to attain hyperactivated motility in the female reproductive tract (Dcunha et al. 2022), the same must be true for the in vitro environment. Therefore, the 2 mM implement for red‐rumped agouti sperm was enough to trigger a good HYP after capacitation.

## 5 Conclusion

The findings indicate that, during the in vitro capacitation of epididymal spermatozoa from the red‐rumped agouti, the presence of CaCl₂ is indispensable for maintaining cell viability and functionality, while also reducing ROS production and achieving high rates of capacitation, acrosome reaction, and sperm hyperactivity. Moreover, a low concentration of BSA at 4 mg/mL proved to be the most effective in maintaining sperm morphology and enhancing capacitation outcomes. A 6‐h incubation seems to be the most suitable for this species, particularly when combined with the LBCa medium, which promoted the highest capacitation and acrosome reaction rates. Further research is essential to determine whether these capacitated spermatozoa possess enhanced fertilizing capacity with oocytes during IVF. This study represents the first comparative analysis of in vitro sperm capacitation supplementations in the red‐rumped agouti, marking a significant advance in understanding the species' sperm physiology and contributing to the refinement of ARTs in wild rodents.

## Author Contributions

**Lhara Ricarliany Medeiros Oliveira:** methodology, investigation, writing – original draft, conceptualization. **Leonardo Vitorino Costa Aquino:** methodology, investigation. **Antonia Beatriz Mendonça Pereira:** methodology, investigation. **Ana Lívia Rocha Rodrigues:** methodology, investigation. **Luanna Lorenna Vieira Rodrigues:** methodology, investigation. **Luana Grasiele Pereira Bezerra:** methodology. **Romário Parente dos Santos:** formal analysis. **Moacir Franco Oliveira:** investigation, methodology. **Alexandre Rodrigues Silva:** writing – review and editing, investigation. **Alexsandra Fernandes Pereira:** investigation, conceptualization, writing – original draft, writing – review and editing, formal analysis, project administration, data curation, supervision, resources.

## Conflicts of Interest

The authors declare no conflicts of interest.

## Data Availability

The data supporting this study's findings are available from the corresponding author upon reasonable request.
